# Metabolomics: Uncovering Insights into Obesity and Diabetes

**DOI:** 10.3390/ijms26136216

**Published:** 2025-06-27

**Authors:** Mansor Fazliana, Tikfu Gee, Shu Yu Lim, Poh Yue Tsen, Zubaidah Nor Hanipah, Nur Azlin Zainal Abidin, Tan You Zhuan, Farah Huda Mohkiar, Liyana Ahmad Zamri, Haron Ahmad, Mohd Shazli Draman, Noorizatul Syahira Yusaini, Mohd Naeem Mohd Nawi

**Affiliations:** 1 Nutrition, Metabolism and Cardiovascular Research Centre, Institute for Medical Research, National Institutes of Health, Ministry of Health Malaysia, Setia Alam, Shah Alam 40170, Selangor, Malaysia; 2 iHEAL Medical Centre, Mid Valley City, Kuala Lumpur 59200, Malaysia; 3 Sunway Medical Centre Velocity, Kuala Lumpur 55100, Malaysia; 4 Sunway Medical Centre, Subang Jaya 47500, Selangor, Malaysia; 5 Department of Surgery, Faculty of Medicine and Health Sciences, University Putra Malaysia, Serdang 43400, Selangor, Malaysia; 6 KPJ Damansara Specialist Hospital, Damansara Utama, Petaling Jaya 47400, Selangor, Malaysia

**Keywords:** obesity, diabetes, prediabetes, metabolomics, ^1^H-NMR, BMI

## Abstract

Obesity is a complex, diverse, and multifactorial disease that has become a significant public health concern. It is a modifiable risk factor for developing type 2 diabetes (T2D). The current classification systems rely on anthropometric measurements, such as body mass index (BMI), which cannot capture the physiopathological diversity of this disease. This study aimed to analyze the metabolic signatures of obesity and diabetes using ^1^H-nuclear magnetic resonance (NMR). Obese patients with BMI ≥ 25 kg/m^2^ (according to the Asian cut-off value) with different diabetes status scheduled to undergo metabolic-bariatric surgery at three hospitals were prospectively recruited for this study. Plasma samples of 111 obese patients and 26 healthy controls were analyzed by ^1^H-NMR. When compared among groups with different diabetes statuses, four clusters with no differences in BMI but different metabolomics profiles were obtained. These clusters highlight intricate metabolic relationships associated with obesity and diabetes. This study demonstrated the benefits of using precision techniques like ^1^H-NMR to better early detection, substantially decreasing the risk of developing T2D and its related complications. This study is the first to report on metabolic markers and altered metabolic profiles of T2D and prediabetes among obese Malaysians with a BMI cut-off value for the Asian population.

## 1. Introduction

Obesity is a major, modifiable risk factor for developing type 2 diabetes (T2D), and nearly 90% of people with T2D are overweight or obese [1]. Excess body fat, especially abdominal fat, can disrupt the body’s metabolic processes, leading to insulin resistance and raised blood sugar levels [2,3]. Eventually, the pancreas may struggle to produce enough insulin, leading to high blood sugar levels and T2D [1]. Obesity also can lead to metabolic changes, including changes in blood sugar, lipid levels, and blood pressure, which increase the risk of heart disease and stroke [4]. Furthermore, obesity is associated with chronic inflammation, which can lead to blood vessel damage and insulin resistance [5]. Addressing obesity and weight management can assist, prevent, delay, and manage the progression of diabetes [1,6,7].

It has emerged as a major global health issue, where 537 million people worldwide are currently affected by diabetes as estimated by the International Diabetes Federation (IDF). By 2030, this number is expected to increase to 578 million, and by 2045, it will reach 783 million, reflecting a 46% rise over the next two decades [8]. Malaysia exhibits one of the highest prevalences of diabetes within the Western Pacific region [9]. The pooled prevalence of diabetes in Malaysia is at 14.39% (95% CI, 12.51–16.38%) among adults aged 18 and older, derived from 15 studies with a total of 103,063 participants [10].

The prevalence of diabetes in Malaysia has significantly increased over the years. The National Health and Morbidity Survey 2023 indicated that 15.6% of adults aged 18 and older were diagnosed with diabetes [11].

Multiple factors influence the onset of T2D, with genetic variants contributing, albeit to a limited extent. As of 2023, around 700 genetic variants linked to a higher risk of T2D have been discovered through genome-wide association studies (GWASs) [12]. Early intervention in T2D is essential to avert long-term complications, including kidney failure, heart disease, and blindness. Relying exclusively on traditional clinical risk factors such as fasting blood glucose, body mass index (BMI), glycated hemoglobin (HbA1c), and blood pressure may prove inadequate for early and precise prediction. Conventional risk factors, although beneficial, possess inherent limitations. Fasting blood glucose and HbA_1_c indicate glucose metabolism issues; however, they may not encompass the complete range of metabolic dysfunctions before the onset of T2D [13]. BMI and blood pressure are significant indicators; however, they do not offer a comprehensive understanding of the underlying metabolic changes [14,15]. Metabolomics, which examines the entire array of small-molecule metabolites in a biological system, provides a more thorough method for predicting T2D [16,17,18]. Metabolomic research has revealed particular serum metabolites linked to a heightened risk of T2D. Metabolites like blood acylcarnitines, branched-chain amino acids (BCAAs), and specific choline-containing phospholipids are included [19]. Models utilizing metabolomic data for machine learning demonstrate superior predictive accuracy for future T2D incidence compared to those relying exclusively on clinical risk factors [20]. A study with 543 non-diabetic participants demonstrated that a novel biomarker panel identified via metabolomics enhanced predictive performance [21]. Early detection via metabolomics facilitates timely interventions, substantially decreasing the risk of developing T2D and its related complications, evidenced by an area under the receiver operating characteristic curve (AUC) of 0.78, which is notably superior to the reference model based solely on clinical risk factors (AUC = 0.68) [21]. This integrated approach underscores the significance of metabolomics in improving the prediction and prevention of T2D, thereby reducing its economic and health impacts.

Nuclear magnetic resonance (NMR) is one of the main methods used in metabolomics, alongside gas chromatography-mass spectrometry (GC-MS) and liquid chromatography-mass spectrometry (LC-MS). NMR spectroscopy is used in diabetes metabolomics studies because it provides a non-destructive and quantitative method for analyzing metabolites in biological fluids, like plasma, serum, and urine. NMR offers advantages in terms of reproducibility, sample preparation simplicity, and the ability to handle diverse biofluids, making it suitable for large-scale studies [22,23].

It would be interesting how these metabolites are associated with our study groups. For example, trimethylamine N-oxide (TMAO), a metabolite produced from specific nutrients by the liver and gut bacteria, is increasingly studied for its connection to diabetes and related complications, with NMR spectroscopy having an important role in its measurement. While some studies suggest TMAO is linked to increased diabetes risk and complications, others indicate potential protective effects, and the overall role of TMAO in diabetes remains controversial [24]. Glycine, in particular, has been linked to insulin sensitivity and resistance, with lower glycine levels often observed in individuals with diabetes [25]. How these metabolites are associated with our study groups would be interesting.

Our study seeks to analyze the metabolic signatures of obesity and diabetes using ^1^H nuclear magnetic resonance (NMR) metabolomics, aiming to identify specific patterns that can aid in early diagnosis, risk prediction, and personalized treatment strategies independent of traditional clinical risk factors.

## 2. Results

### 2.1. Participants’ Characteristics

Table 1 presents the general characteristics of the 137 study participants. The bariatric surgery patients were stratified based on their HbA_1_c levels into three groups: obese non-diabetes, obese with prediabetes, and obese with diabetes. Additionally, 26 lean, healthy individuals were recruited as controls. The mean age of the participants was 36.3 (±9.1) years, and the majority were female (63.5%). The mean BMI was significantly higher in all obese groups compared to the healthy controls. Among the obese groups, those with prediabetes and diabetes had significantly higher BMI than the non-diabetes group (Table 1). Additionally, several metabolic risk factors, including HbA1c, triglycerides (TG), and HDL-C, showed significant differences across the groups. HbA1c and TG levels were highest, while HDL-C levels were lowest in the obese diabetes group, following a stepwise increase from healthy controls to obese non-diabetes, obese prediabetes, and obese diabetes. There were no significant differences in total cholesterol (TC) and LDL-C levels across the groups.

### 2.2. Overall Analysis

**Figure 1:**
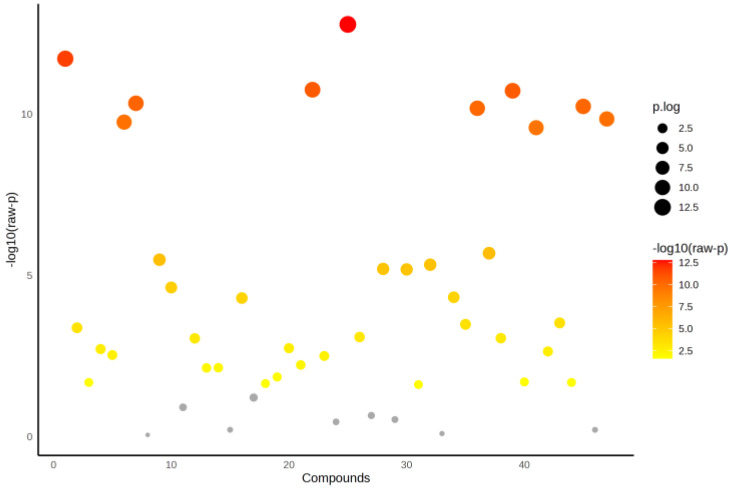
ANOVA for all groups (Healthy control, obese only, obese prediabetes, obese diabetes).

The ^1^H NMR spectra are illustrated in Supplementary Figures S1 and S2. ANOVA was carried out to identify metabolites that differed significantly between groups, i.e., obese only, obese prediabetes, obese diabetes vs. healthy control group. Figure 1 represents the distribution of metabolites based on their statistical significance, expressed as −log10(p). Overall, 47 metabolites were detected (Table S1). Among the analyzed metabolites, ten were highly significant (*p* < 0.001, Table 2) while 38 showed significant differences (*p* < 0.05). Nine metabolites were not significantly different (*p* > 0.05) and are depicted in gray. The color scale, ranging from yellow to red, reflects the level of significance, with red indicating the most statistically significant metabolites. Additionally, the size of each data point represents the magnitude of the effect, providing a visual representation of key metabolic alterations.

### 2.3. Fold Change, T-Test, and Volcano Plot Relative to Healthy Controls

(i) Obese only

**Figure 2:**
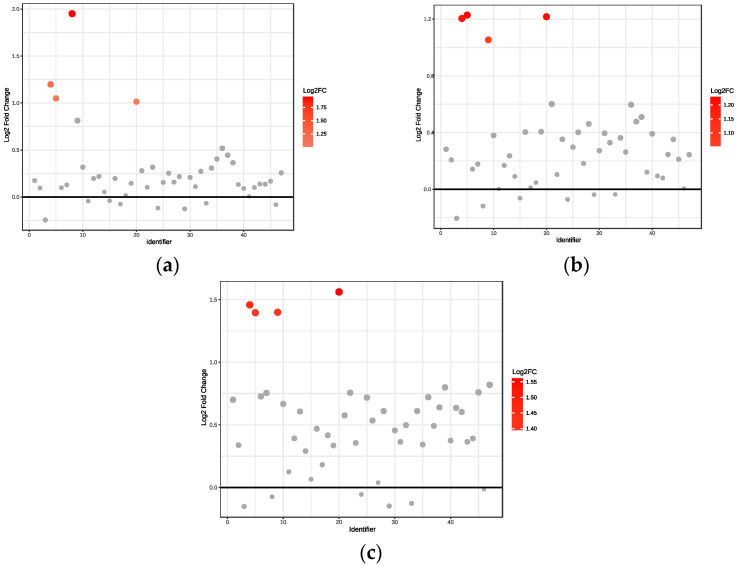
Fold change relative to healthy controls: (**a**) Obese only, (**b**) Obese prediabetes, (**c**) Obese diabetes. Black horizontal line: Indicates the reference value for Log2 Fold Change (usually set at 0), representing no change between groups. Points above this line have increased expression (or abundance), and points below would have decreased expression, relative to the reference group.

We observed significant upregulation of four metabolites in obese non-diabetes patients compared to healthy controls (Figure 2a). Specifically, acetate had a 3.9-fold increase, whereas 3-aminoisobutyrate, 3-hydroxybutyrate, and fucose had a 2.0–2.3-fold increase.

**Figure 3:**
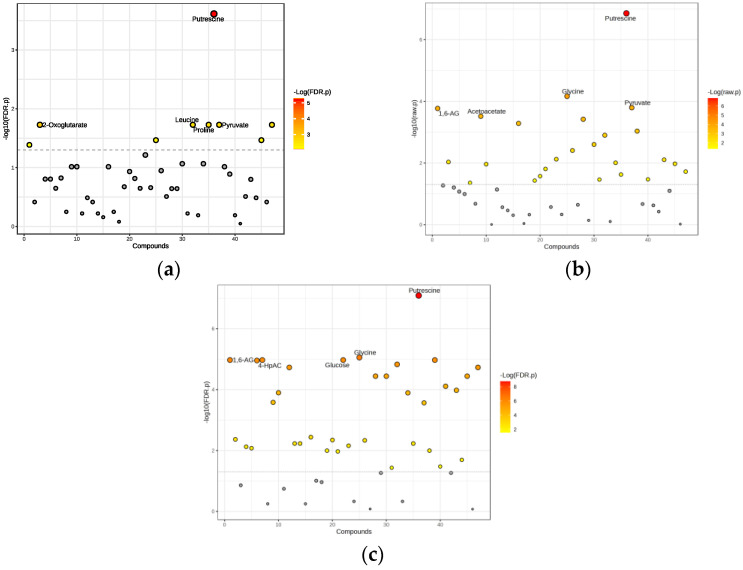
Significant metabolites differentiating the obese diabetes group from healthy controls: (**a**) Obese only, (**b**) Obese prediabetes, (**c**) Obese diabetes. Gray dots: Represent features (such as metabolites or genes) that do not meet the threshold for significant change. These are considered not significantly different between groups, in contrast to the colored (red) dots, which indicate significant changes. 1,6-AG—1,6-Anhydro-β-D-glucose; 4-HpAC—4-Hydroxyphenylacetate.

We identified nine significant metabolites differentiating the obese non-diabetes group from healthy controls (Figure 3a). Significantly, the metabolites with the highest significance levels included putrescine, 2-oxoglutarate, leucine, proline, and pyruvate.

**Figure 4:**
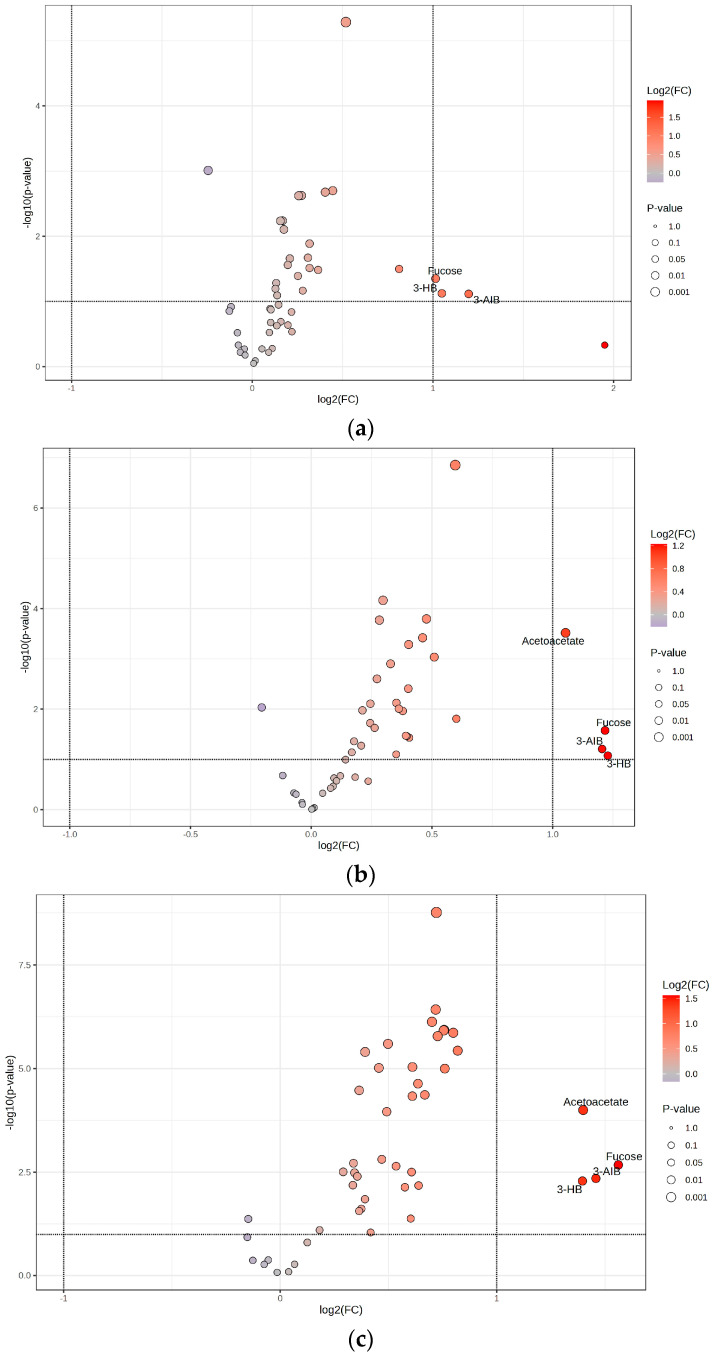
Volcano plots for (**a**) Obese only, (**b**) Obese prediabetes, (**c**) Obese diabetes. Horizontal dotted line: Represents the significance cutoff for the p-value (commonly set at *p* = 0.05). Points above this line have *p*-values lower than the cutoff and are considered statistically significant. Vertical dotted lines: Represent the fold change thresholds (commonly log2(FC) = ±1, which corresponds to a 2-fold change). Points to the right or left of these lines have fold changes greater than or less than the set threshold, indicating substantial upregulation or downregulation. 3-AIB—3-Aminoisobutyrate; 3-HB—3-Hydroxybutyrate.

To illustrate these results, a volcano plot was generated, depicting the correlation between fold change and statistical significance (Figure 4a). The volcano plot illustrates the relationship between log2 fold change (*x*-axis) and the negative log10 of the *p*-values (*y*-axis), with metabolites, namely 3-hydroxybutyrate, 3-aminoisobutyrate, and fucose in the upper regions indicating high statistical significance and their horizontal position reflecting the magnitude of change.

(ii) Obese prediabetes

Significant upregulation of four metabolites was observed in obese prediabetes patients compared to healthy controls (Figure 2b). Specifically, 3-hydroxybutyrate, fucose, and 3-aminoisobutyrate exhibited a 2.3-fold increase, indicating their concentration is 2.3 times higher in the prediabetic group, while acetoacetate had a 2-fold increase.

A total of 30 significant metabolites were identified, with *p* < 0.05 indicating statistical significance (Figure 3b). Noteworthy metabolites with the highest significance included putrescine, glycine, pyruvate, 1,6-anhydro-D-glucose, and acetoacetate. The relationship between fold change and statistical significance is visualized by a volcano plot (Figure 4b).

The volcano plot effectively highlights key metabolites that warrant further investigation into their roles in metabolic pathways and their potential as therapeutic targets. Metabolites such as hydroxybutyrate, fucose, 3-aminoisobutyrate, and acetoacetate in the upper regions indicate high statistical significance, and their horizontal position reflects the magnitude of change.

(iii) Obese diabetes

Our analysis revealed significant upregulation of four metabolites in obese diabetes patients relative to healthy controls (Figure 2c). In particular, fucose demonstrated a 2.95-fold increase, whereas 3-aminoisobutyrate, acetoacetate, and 3-hydroxybutyrate exhibited increases between 2.6- and 2.75-fold.

We conducted a t-test to compare metabolite levels between obese diabetes patients and healthy controls, identifying 37 significant metabolites with *p* < 0.05 (Figure 3c). The metabolites identified as having the highest significance included putrescine, glycine, 1,6-anhydro-D-glucose, glucose, and 4-hydroxyphenylacetate.

A volcano plot was also generated to visualize these differences, illustrating the relationship between fold change and statistical significance for the identified metabolites (Figure 4c). The volcano plot illustrates the relationship between log2 fold change (*x*-axis) and the negative log10 of the *p*-values (*y*-axis), with metabolites fucose, 3-aminoisobutyrate, acetoacetate, and 3-hydroxybutyrate in the upper regions indicating high statistical significance and their horizontal position reflecting the magnitude of change.

### 2.4. PLS-DA All Groups

**Figure 5:**
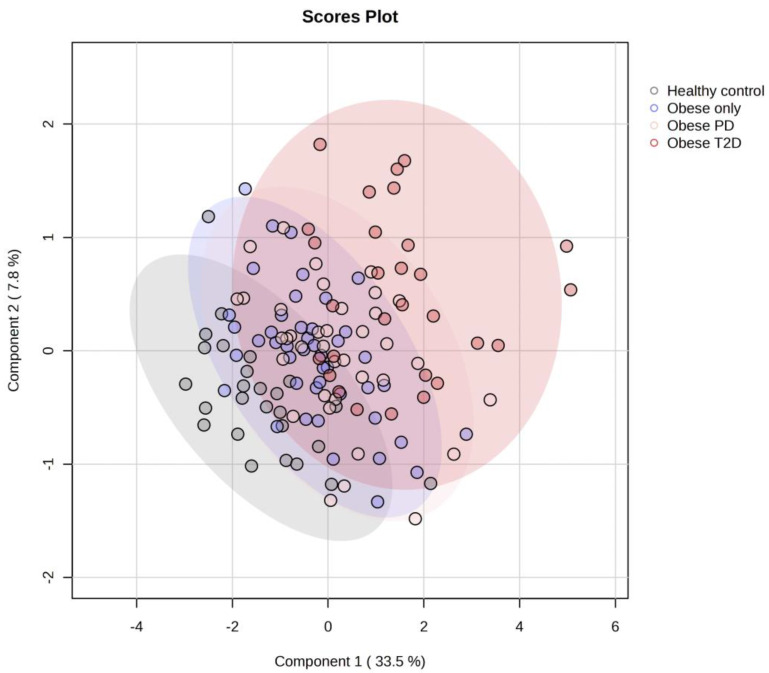
PLS-DA score plot for all four groups. PD—prediabetes; T2D—type 2 diabetes.

The PLS-DA score plot (Figure 5) reveals substantial overlap among the four groups—healthy controls, obese only, obese prediabetes, and obese diabetes—indicating that their metabolic profiles are more similar than distinct.

### 2.5. OPLS-DA

As the use of PLS-DA is less effective when analyzing more than three groups due to increased complexity in interpretation, a higher risk of overfitting, and diminished discriminatory power, we then employed OPLS-DA (Orthogonal Partial Least Squares Discriminant Analysis) with healthy controls as a common reference group to address these issues. This approach allows for a clearer separation of the groups by disentangling group-predictive variation from unrelated variation, thereby enhancing the interpretability of the results and providing a more robust framework for identifying metabolic differences among the groups. These results represent OPLS-DA relative to healthy controls:

(i) Obese only

**Figure 6:**
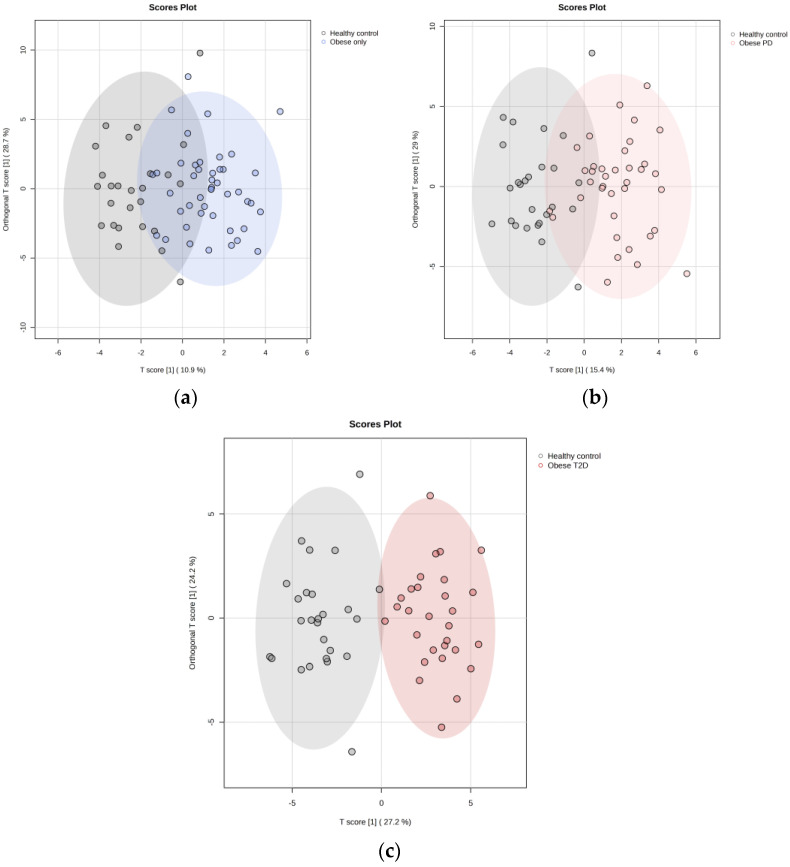
OPLS-DA relative to healthy controls: (**a**) Obese only, (**b**) Obese prediabetes, (**c**) Obese diabetes. PD—prediabetes; T2D—type 2 diabetes.

**Figure 7:**
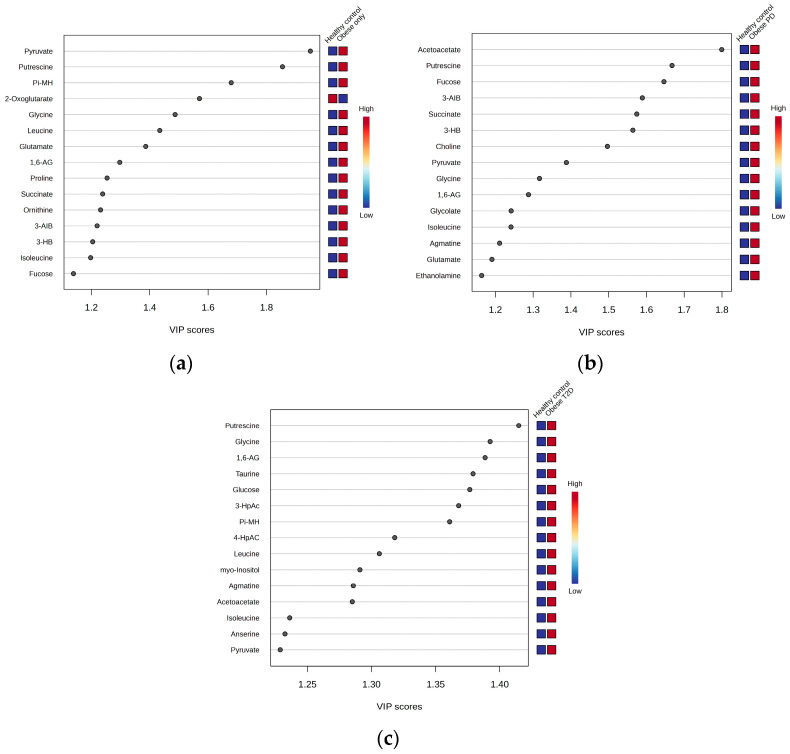
Variable Importance in Projection (VIP) scores: (**a**) Obese only, (**b**) Obese prediabetes, (**c**) Obese diabetes. PD—prediabetes; T2D—type 2 diabetes; 1,6-AG—1,6-Anhydro-β-D-glucose; 3-AIB—3-Aminoisobutyrate; 3-HB—3 Hydroxybutyrate; 3-HpAC—3-Hydroxyphenylacetate; 4-HpAC—4-Hydroxyphenylacetate; Pi-MH—π-methylhystidine.

We employed OPLS-DA to assess metabolic differences between obese only patients and healthy controls (Figure 6a). The model demonstrated a cumulative R^2^Y value of 0.337, indicating that 33.7% of the variance in the metabolite data was explained by group membership, along with a Q^2^ value of 0.235, suggesting reasonable predictive ability. The analysis identified five key metabolites with the highest Variable Importance in Projection (VIP) scores (Figure 7a): pyruvate, putrescine, π-methylhistidine, 2-oxoglutarate, and glycine. Notably, all these metabolites, except for 2-oxoglutarate, were upregulated in the obese only group compared to healthy controls. This distinct metabolic profile underscores the significance of our research, as it highlights potential biomarkers for obesity and emphasizes the complexity of metabolic interactions within these populations.

(ii) Obese prediabetes

We also utilized OPLS-DA to evaluate the metabolic differences between obese prediabetes patients and healthy controls (Figure 6b). The model demonstrated a cumulative R^2^Y value of 0.4, indicating that 40% of the variance in the metabolite data could be explained by group membership, suggesting a moderate level of model fit. The Q^2^ value of 0.318 reflects the model’s predictive ability, indicating reasonable predictive power, although there is room for improvement. The analysis identified five key metabolites with the highest VIP scores (Figure 7b): acetoacetate, putrescine, Fucose, 3-aminoisobutyrate, and succinate. Notably, these metabolites were found to be upregulated in obese prediabetes patients compared to healthy controls.

(iii) Obese diabetes

OPLS-DA is again used to assess metabolic differences between obese diabetes patients and healthy controls (Figure 6c). The model demonstrated a cumulative R^2^Y value of 0.57, indicating that 57% of the variance in the metabolite data was explained by group membership, along with a Q^2^ value of 0.547, suggesting strong predictive ability. The analysis identified five key metabolites with the highest VIP scores (Figure 7c): putrescine, glycine, 1,6-Anhydro-D-glucose, taurine, and glucose. Notably, all these metabolites were upregulated in the obese diabetes group compared to healthy controls. Putrescine is involved in cellular growth, while taurine plays a role in various physiological processes, underscoring their significance in metabolic pathways. Distinct metabolic profiles were observed between the two groups, although some overlap indicated shared metabolic features. Overall, our findings suggest that OPLS-DA effectively elucidates metabolic differences, highlighting potential biomarkers for obesity-related diabetes and emphasizing the complexity of metabolic interactions within these populations.

### 2.6. Hierarchal Clustering Analysis (HCA)

**Figure 8:**
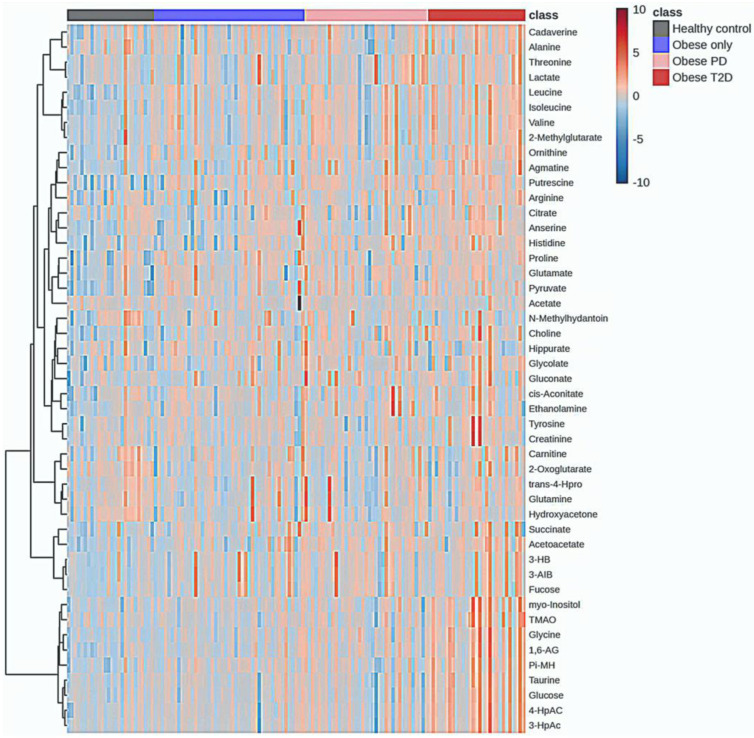
Hierarchal Clustering Analysis (HCA). PD—prediabetes; T2D—type 2 diabetes; 1,6-AG—1,6-Anhydro-β-D-glucose; 3-AIB—3-Aminoisobutyrate; 3-HB—3 Hydroxybutyrate; 3-HpAC—3-Hydroxyphenylacetate; 4-HpAC—4-Hydroxyphenylacetate; trans-4-Hpro—trans-4-Hydroxy-L-proline; TMAO—Trimethylamine N-oxide; Pi-MH—π-methylhystidine.

Hierarchical clustering analysis was conducted to explore the metabolic profiles of healthy controls, obese only, obese prediabetes, and obese diabetes (Figure 8).

The analysis revealed significant metabolic differences among these groups. Healthy controls exhibited distinct metabolic signatures compared to the obese groups; however, some subjects within the healthy control group showed an overlap in metabolic characteristics with the obese groups. These individuals were identified as outliers, indicating that while they generally belong to the healthy control group, their metabolic profiles resemble those of individuals with obesity.

Notably, there was some overlap in metabolic characteristics between the obese only and obese prediabetes groups, while the obese diabetes group displayed unique metabolic alterations associated with diabetes. The accompanying heatmap visually represented metabolite expression levels across the groups, indicating that certain metabolites were consistently upregulated in the obese diabetes group compared to the others.

**Figure 9:**
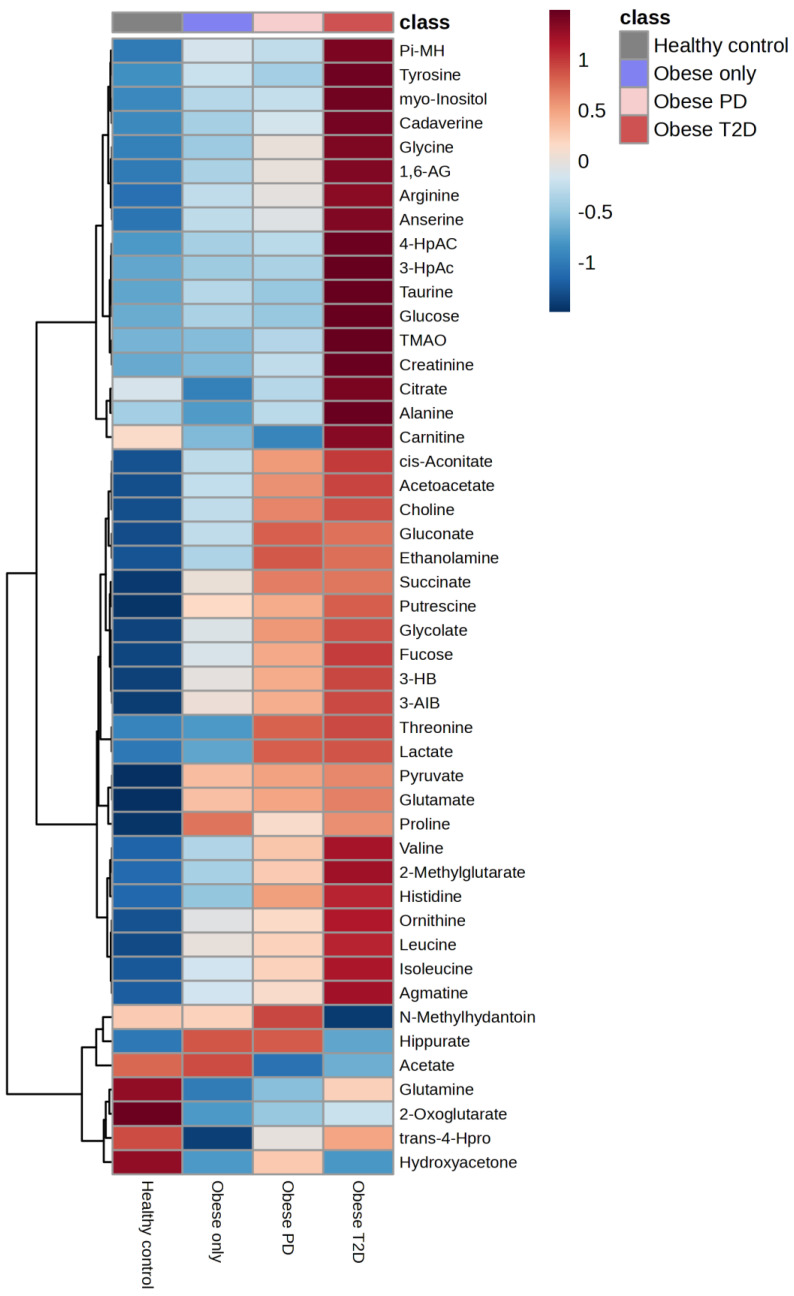
The integration of the average results from hierarchical clustering analysis. PD—prediabetes; T2D—type 2 diabetes; 1,6-AG—1,6-Anhydro-β-D-glucose; 3-AIB—3-Aminoisobutyrate; 3-HB—3 Hydroxybutyrate; 3-HpAC—3-Hydroxyphenylacetate; 4-HpAC—4-Hydroxyphenylacetate; trans-4-Hpro—trans-4-Hydroxy-L-proline; TMAO—Trimethylamine N-oxide; Pi-MH—π-methylhystidine.

The integration of the average results from HCA reveals a distinct group of metabolites that are massively upregulated in the obese diabetes group (Figure 9), including putrescine, glycine, 1,6-Anhydro-D-glucose, taurine, glucose, tyrosine, myoinositol, cadaverine, 3-hydroxyphenylacetate, 4-hydroxyphenylacetate, TMAO, creatinine, and alanine.

Conversely, several metabolites, including succinate, putrescine, amino isobutyrate, pyruvate, and glutamate, were strongly downregulated in the healthy control group.

**Figure 10:**
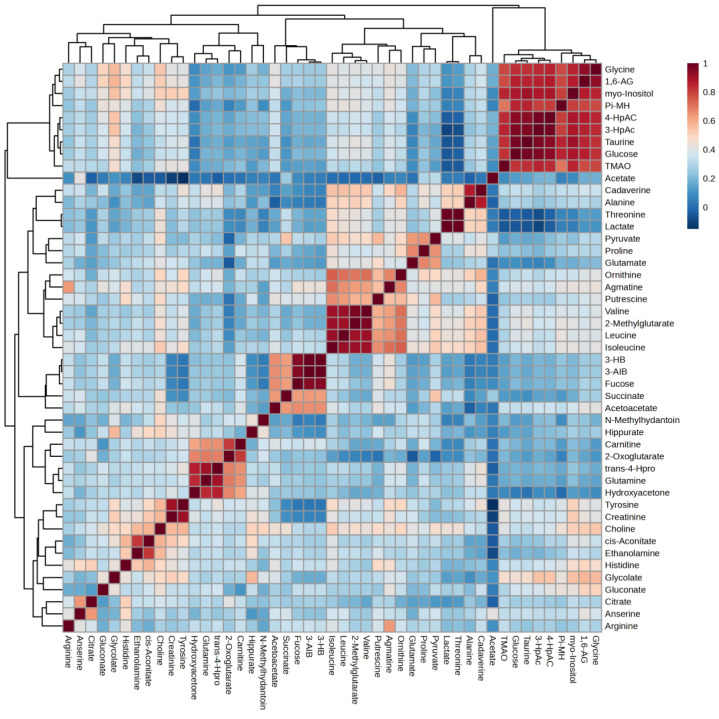
Correlation heatmap. 1,6-AG—1,6-Anhydro-β-D-glucose; 3-AIB—3-Aminoisobutyrate; 3-HB—3 Hydroxybutyrate; 3-HpAC—3-Hydroxyphenylacetate; 4-HpAC—4-Hydroxyphenylacetate; trans-4-Hpro—trans-4-Hydroxy-L-proline; TMAO—Trimethylamine N-oxide; Pi-MH—π-methylhystidine.

Correlation heatmap of this study is presented in Figure 10. Glycine, 1,6-anhydro-D-glucose, myo-inositol, π-methylhistidine, 4-hydroxyphenylacetate, 3-hydroxyphenylacetate, taurine, glucose, and TMAO form Cluster 1, characterized by strong positive correlations with one another. This clustering suggests that these metabolites may be interconnected within metabolic pathways, indicating their potential collective role in the metabolic profile observed in our study. Additionally, several smaller clusters were identified: Cluster 2, comprising valine, 2-methylglutarate, leucine, and isoleucine; Cluster 3, consisting of 3-hydroxybutyrate, 3-aminoisobutyrate, and fucose; and Cluster 4, which includes trans-4-hydroxy-L-proline, glutamine, and hydroxyacetone.

Each cluster also exhibited strong correlations among their respective metabolites, further highlighting the intricate relationships within the metabolic network associated with obesity and diabetes. We will discuss these four clusters further in the Discussion session.

## 3. Discussion

Our study, to our knowledge, is the first to unveil predictive metabolic markers and altered metabolic profiles of T2D and prediabetes among obese individuals in Malaysia with a BMI cut-off value for the Asian population.

The strong overlap among the four groups in the OPLS-DA, particularly among the obese categories, highlights the complexity of metabolic responses and indicates that differentiating between these conditions based solely on metabolite profiles may be challenging, underscoring the need for further research in this area. While distinct metabolic profiles were observed between the obese prediabetes group and healthy controls, some overlap indicated that specific metabolites may not be exclusively altered in one group. This overlap suggests that while there are clear metabolic disturbances associated with obesity and prediabetes, there may also be shared metabolic features present in healthy individuals. Overall, our findings suggest that OPLS-DA is an effective method for elucidating metabolic differences between these groups, providing valuable insights into the underlying pathophysiological mechanisms contributing to obesity-related conditions. The identification of these key upregulated metabolites not only highlights potential biomarkers for obesity and prediabetes but also emphasizes the complexity of metabolic interactions within these populations.

Putrescine, one of the metabolites upregulated in the obese diabetes group compared to healthy controls, is involved in cellular growth, while taurine plays a role in various physiological processes, underscoring their significance in metabolic pathways. Distinct metabolic profiles were observed between the two groups, although some overlap indicated shared metabolic features. Overall, our findings suggest that OPLS-DA effectively elucidates metabolic differences, highlighting potential biomarkers for obesity-related diabetes and emphasizing the complexity of metabolic interactions within these populations.

For HCA, our finding reinforces the notion of metabolic dysregulation in obesity-related diabetes. Overall, these results from hierarchical clustering analysis emphasize the metabolic distinctions between healthy individuals and various stages of obesity and diabetes, while highlighting potential biomarkers for further investigation into obesity-related metabolic disturbances. Significant upregulation in the obese diabetes group, alongside the downregulation observed in healthy controls, underscores the metabolic alterations associated with obesity and diabetes, providing insights into potential biomarkers for further investigation into obesity-related metabolic disturbances.

The correlation heatmap analysis has unveiled four distinct metabolite clusters, providing a comprehensive view of the metabolic interactions across all study groups. This approach, in line with our study’s aim, offers a more integrated analysis than individual analytical methods. Focusing on the correlation heatmap can provide a more holistic interpretation of the metabolomic data, offering insights into the complex metabolic alterations associated with obesity and diabetes in the Malaysian population. These findings could potentially revolutionize diabetes management strategies.

Through the correlation heatmap, four clusters were formed. The most significant cluster, Cluster 1: Glycine, 1,6-anhydro-D-glucose, myo-inositol, methylhistidine, 4-hydroxyphenylacetate, 3-hydroxyphenylacetate, taurine, glucose, and trimethylamine N-oxid (TMAO), characterized by strong positive correlations with one another. This clustering suggests that these metabolites may be interconnected within metabolic pathways, indicating their potential collective role in the metabolic profile observed in our study. Additionally, several smaller clusters were identified: Cluster 2, comprising valine, 2-methylglutarate, leucine, and isoleucine. Next, Cluster 3, consisting of 3-hydroxybutyrate, 3-amino isobutyrate, and fucose; and finally, Cluster 4, which includes trans-4-hydroxy-L-proline, glutamine, and hydroxyacetone. Each cluster also exhibited strong correlations among their respective metabolites, further highlighting the intricate relationships within the metabolic network associated with obesity and diabetes. We summarize these metabolites in Table 3. Some of the metabolites will be discussed below.

### 3.1. Cluster 1

#### 3.1.1. Glycine

Glycine has been suggested as a conditionally essential amino acid. In metabolic disorders associated with obesity, T2D, and non-alcoholic fatty liver disease (NAFLD), lower circulating glycine levels have been consistently observed. Glycine supplementation has beneficial effects, as suggested in clinical studies [26]. Individuals with obesity and T2D tend to have lower glycine levels, suggesting that glycine depletion might be a factor in the development of diabetes [19].

#### 3.1.2. Myo-Inositol

Myo-inositol, a type of inositol, has shown promise in improving cardiometabolic factors, including lipid profiles and liver function, and reducing BMI in obese individuals, potentially through mechanisms like improved insulin sensitivity and thyroid function [27,28]. Myo-inositol was shown to possess insulin-mimetic properties and efficiently lower post-prandial blood glucose [29]. In an animal model study, abnormalities in myo-inositol metabolism are associated with T2D in mice fed a high-fat diet [30]. Myo-inositol urinary excretion was strongly correlated with glucose urinary excretion. Interestingly, some research suggests that myo-inositol supplementation may help prevent gestational diabetes mellitus in overweight and obese pregnant women [31]. A recent large-scale cohort study of obese individuals in China found a potential obesity-linked bacterium, Megamonas. This research suggests potential strategies for future obesity management by illustrating how the bacterium degrades intestinal myo-inositol, enhances lipid absorption, and contributes to obesity [32].

#### 3.1.3. Trimethylamine N-Oxide (TMAO)

TMAO, a gut microbiota-derived metabolite, has been associated with an elevated risk of obesity and cardiovascular disease, potentially acting as a novel risk factor. Studies suggest that elevated TMAO levels are associated with obesity and related metabolic issues, such as insulin resistance and increased risk of cardiovascular disease [33,34]. TMAO’s role in metabolic pathways and its impact on gut microbiota composition may contribute to the progress of obesity and related metabolic disorders [35]. The composition of the diet, particularly the TMAO precursors intake like choline, carnitine, and betaine, can influence TMAO levels. Interestingly, elevated levels of serum TMAO levels were positively associated with the risk of diabetic kidney disease in T2D patients [36].

#### 3.1.4. Taurine

Metabolomic studies have shown that taurine supplementation can improve lipid metabolism and reduce the risk of metabolic syndrome in people with obesity. Taurine may also help prevent and manage T2D [37,38]. Taurine administration prevented weight gain and dyslipidemia, and alleviated lipid deposition in the liver and adipose tissue in hyperlipidemic mice [39]. Furthermore, taurine reduces the risk of metabolic syndrome [40].

### 3.2. Cluster 2

#### Branched-Chain Amino Acids (BCAAs)

BCAAs are essential amino acids that change along with consuming a protein-containing meal, including valine, leucine, and isoleucine [41]. Circulating BCAAs have emerged as strongly and positively associated with adiposity [42]. Studies have also shown a positive association between increased circulating BCAAs (valine, leucine, and isoleucine) and insulin resistance (IR) in obese or diabetic patients [43]. Isoleucine and valine levels are higher among the obese patients with T2D compared to healthy individuals [44,45]. In addition, BCAA metabolites’ downstream accumulation may cause mitochondrial dysfunction [46]. Meanwhile, elevated BCAA levels are associated with insulin resistance [47], diabetes [48], and coronary artery disease [49] However, the physiological mechanisms underlying the regulation of circulating BCAA concentrations remain unknown [50,51,52,53]. There are two potential mechanisms explaining how BCAAs might contribute to insulin resistance in T2D and obesity [46]. First, excess dietary BCAAs activate the mammalian rapamycin complex 1 (mTORC1) signaling target, leading to insulin resistance and T2D. Second, it has been suggested that increased BCAAs could serve as a practical biomarker of impaired metabolism, offering potential for clinical applications.

### 3.3. Cluster 3

#### 3.3.1. 3-Hydroxybutyrate (3HB)

3HB, a ketone body, has gained attention for its potential role in obesity and related metabolic disorders, with research suggesting it may influence adipocyte function and gut microbiota and potentially reduce insulin resistance [54,55]. It has been shown that the 3HB ameliorates insulin resistance by inhibiting PPARγ Ser273 phosphorylation in T2D mice [56]. 3HB levels increase in people with diabetes and prediabetes [57].

#### 3.3.2. 3-Amino Isobutyrate

3-amino isobutyrate (also known as β-aminoisobutyric acid or BAIBA) is a catabolic metabolite of thymine and valine in skeletal muscle [58,59]. During exercise, BAIBA is released by contracting human skeletal muscle and lowers insulin release from INS-1 832/3 cells by mediating mitochondrial energy metabolism. It is a possible mediator of insulin secretion [60]. During exercise, 3-aminoisobutyric acid is secreted by muscle cells into the blood. Once this molecule interacts with adipocytes, it may induce signaling pathways regulating fats, insulin, and cholesterol metabolism.

#### 3.3.3. Fucose

L-fucose, the main form of fucose found in nature, has shown promising anti-obesity effects, potentially by suppressing lipid accumulation and improving insulin sensitivity, and may be a novel strategy for treating obesity and related diseases. L-fucose suppresses lipid accumulation via the AMPK Pathway in 3T3-L1 adipocytes [61]. L-fucose can improve the gut microbiota’s obesity-driven dysbiosis, potentially by decreasing the relative abundance of obesity-related intestinal bacteria [62]. Fucose might be a novel strategy to treat HFD-induced obesity and fatty liver [63]. 

### 3.4. Cluster 4

#### 3.4.1. trans-4-Hydroxy-L-proline

While there is no direct, strong scientific evidence linking trans-4-hydroxy-L-proline (t4Hyp) to obesity, it is a valuable chiral building block used in pharmaceuticals and cosmetics, and its production is being explored through metabolic engineering. However, some patents mention t4Hyp as a potential anti-obesity agent, though this is not widely supported by scientific evidence (US Patent application No. US20060264498A1) [64].

#### 3.4.2. Glutamine

Research suggests a link between glutamine, a non-essential amino acid, and obesity, with studies indicating that glutamine metabolism is altered in obese individuals and may play a role in inflammation within fat tissue, potentially impacting obesity-related conditions [65]. Some studies suggest that glutamine supplementation may reduce obesity and pro-inflammatory markers and improve insulin sensitivity in animal models and overweight/obese humans. Oral glutamine supplementation reduces obesity, and pro-inflammatory markers, improves insulin sensitivity in rats, and reduces waist circumference in overweight and obese human [66].

#### 3.4.3. Hydroxyacetone

Hydroxyacetone (acetol) is a metabolite of acetone found in people with diabetic ketoacidosis [67]. It can bind to and modify proteins, which may contribute to the progress of diabetes complications. Hydroxyacetone is an organic compound that can be produced by reducing methylglyoxal. Diets rich in vegetables, whole grains, fruits, and lean protein contribute to normal metabolism and low levels of methylglyoxal production. In stark contrast, a western diet of processed food, refined grains, and red meat leads to metabolic dysfunction, increasing methylglyoxal levels, and is associated with several pathologies, including obesity, heart disease, diabetes, and cancer [68]

**Table 3:** Summary table of metabolites identified from four clusters.

Metabolites	Author	Populations	Platform	Findings
Cluster 1				
Glycine	Thalacker-Mercer, A.E., et al. [69]	124 adults (60 African American and 63 European American)	Hyperinsulinemic-euglycemic clamp	Concentration of glycine was correlated to glucose disposal rate (GDR)
	Cheng, S., et al. [70]	Framingham Heart Study (n = 1015); Malmö Diet & Cancer Study (n = 746)	Liquid chromatography-tandem mass spectrometry (LC-MS)	The criteria for metabolic syndrome were met by 45% individuals
	Floegel, A., et al. [19]	(EPIC)-Potsdam study (2282 individuals of the subcohort and 800 individuals with incident T2D)	Tandem mass spectrometry (FIA-MS/MS)	Glycine levels tend to be lower in individuals with obesity and T2D
	Takashina, C., et al. [71]	Ninety-four healthy Japanese volunteers aged 20–60 years	HPLC	The 2 h or fasting plasma glucose levels or HOMA-IR were positively correlated with glutamate, valine, and tyrosine levels but negatively correlated with glutamine, citrulline, and glycine levels
	Tulipani, S., et al. [72]	64 adults; sex-matched groups for BMI and risk of developing T2D	LC- and flow injection analysis-(electrospray ionization) mass spectrometry (FIA-ESI-MS/MS)	Glycine concentration is negatively associated with fasting insulin and HOMA-IR (R = −0.51, R = −0.49, respectively; both *p* < 0.0033)
	Wijayatunga, N.N., et al. [73]	20 patients undergoing RYGB surgery, pre- and 6 months post-surgery	NMR	Serum glycine was significantly elevated at 6 months post-surgery compared with pre-surgery (n = 8, *p* < 0.05)
1,6-anhydro-D-glucose	Kawaguchi, T., et al. [74]	Five male patients with NAFLD; a randomized, single-blinded controlled interventional study	LC-time-of-flight mass spectrometry (TOFMS)	Isomaltulose improved insulin resistance in NAFLD patients
myo-inositol	Croze, M.L., et al. [30]	High-fat diet (HFD) was fed to C57BL/6 male mouse for 1 month (n = 10)	HPLC	Inosituria, which is myo-inositol urinary excretion, was correlated with glycosuria in mice (R^2^ 0.915, linear regression)
	Wu, C., et al. [32]	Chinese cohort of 631 obese subjects and 374 normal-weight controls	Shotgun metagenomic sequencing	Identified a Megamonas-dominated, enterotype-like cluster enriched in obese subjects, where *Megamonas rupellensis* possessed genes for myo-inositol degradation, which is a myo-inositol degrader. This function enhances lipid absorption and obesity
	Sikes, K.J., et al. [75]	90 mice	Gas Chromatography Mass Spectrometry (GCMS)	Untargeted metabolomics analysis identifies creatine, myo-inositol, and lipid pathway modulation in a murine model of tendinopathy
4-hydroxyphenylacetate	Wang, P., et al. [76]	24 mice, 16 weeks	GC-MS	4-HPA was sufficient to reverse obesity and glucose intolerance in HFD-fed mice. Mechanistically, 4-HPA treatment markedly regulates SIRT1 signaling pathways and induces the expression of beige fat and thermogenesis-specific markers in white adipose tissue (WAT)
	Osborn, L.J., et al. [77]	Male C57BL/6 mice	High-performance liquid chromatography tandem mass spectrometry (LC-MS/MS)	a single gut microbial flavonoid catabolite, 4-hydroxyphenylacetic acid (4-HPAA), is sufficient to reduce diet-induced cardiometabolic disease (CMD) burden in mice
	Chen, W., et al. [78]	32 male C57BL mice	Urine metabolomic analysis by UHPLC-Q-TOF/MS	Microbial phenolic metabolites 3-(3′,4′-dihydroxyphenyl) propanoic acid and 3′,4′-dihydroxyphenylacetic acid prevent obesity in mice fed with high-fat diet
3-hydroxyphenylacetate	Zhang, Y., et al. [56]	28 of 6-week-old male ICR/KM mice, high-fat diet	^1^H-NMR	High-fat diet significantly reduced the 3-hydroxyphenylacetate concentrations in the cecum contents of mice
Glucose	Li, M., et al. [79]	Virgin Wistar rats, supplemented with taurine	Autoanalyser	Maternal taurine supplementation may ameliorate the adverse effects observed in offspring following a maternal obesogenic diet
Trimethylamine N-oxid (TMAO)	Zhang, Q., et al. [80]	28 of 6-week-old male ICR/KM mice, high-fat diet	^1^H-NMR	High-fat-diet-induced obese mice supplemented with fibers have lower levels of TMAO, compared to control subjects
	Barrea, L., et al. [34]	330 adult Caucasians subjects (20–63 years) of both genders	HPLC/MS	TMAO levels increased along with BMI and were positively associated with VAI and FLI, independently
	Lee, S.J., et al. [81]	38 obese patients (18 with and 20 without T2D) who underwent bariatric surgery	UPLC/TQ-MS	TMAO increased more than twofold in patients with T2D after surgery, but not in patients without T2D
	Li, X., et al. [82]	Sixteen M. mulatta (six months old) were fed a control diet or a HFHC diet for 18 months	^1^H-NMR	The diet rich in fish affects the concentration of TMAO, also the high-fat and high-calorie diet increases the levels of serum TMAO
**Cluster 2**				
valine	Ho, J.E., et al. [83]	2383 Framingham Offspring cohort participants	LC-MS	Valine was associated with 4 metabolic traits: BMI, HOMA-IR, HDL, and triglycerides; circulating branched-chain amino acids, like valine, have emerged as strongly and positively associated with adiposity
	Tai, E.S., et al. [84]	263 non-obese, Asian-Indian and Chinese men, cross-sectional study in Singapore	Tandem MS (MS/MS)	Insulin resistance (IR) was correlated with increased levels of valine
	Wang, T.J., et al. [48]	The Framingham Offspring Study, 2422, free of diabetes and cardiovascular disease, underwent routine oral glucose tolerance testing (OGTT), >35 years	LC-MS	2422 individuals with normal glucose levels followed for 12 years, 201 developed diabetes Valine had highly significant associations with future diabetes
2-methylglutarate	Wijayatunga, N.N., et al. [73]	20 patients undergoing RYGB surgery pre and 6 months post-surgery	1H-NMR	Serum 2-methylglutarate was significantly reduced, at 6 months post-surgery compared with pre-surgery (n = 8, *p* < 0.05)
Leucine	Ozcariz, E., et al. [85]	1387 individuals from the general population	1H-NMR	Leucine was found to be significantly different between clusters of metabolic profiles of cardiometabolic risk in patients with obesity
Isoleucine	Yu, D., et al. [86]	male C57BL/6J mice	Quadrupole-orbitrap mass spectrometer	Reducing isoleucine or valine rapidly restores metabolic health to diet-induced obese mice. A low isoleucine diet reprograms liver and adipose metabolism, increasing hepatic insulin sensitivity
	Trautman M.E. et al., 2024 [87]	C57BL/6J and DBA/2J mice	Quadrupole-orbitrap mass spectrometer	Reducing dietary levels of isoleucine rapidly improves the metabolic health of diet-induced obese male C57BL/6J mice
**Cluster 3**				
3-hydroxybutyrate (3-OHB)	Zhang, Y., et al. [56]	Four-week-old male C57BL/6 mice	Glucose uptake assay	3OHB improves glucose tolerance, reduces fasting blood glucose level, and ameliorates insulin resistance in T2D mice through hydroxycarboxylic acid receptor 2 (HCAR2)
	Jung, J., et al. [88]	Mice (db/db) were fed normal chow, high-fat, or ketogenic diets	Determination of Reactive Oxygen Species (ROS)	in vivo and in vitro models show that 3-OHB delays the progression of diabetic nephropathy by augmenting autophagy and inhibiting oxidative stress
3-aminoisobutyrate	Yousri, N.A., et al. [89]	996 individuals in Qatar	ultra-performance liquid chromatography (UPLC), mass spectrometer interfaced with a heated electrospray ionization (HESI-II) source and Orbitrap mass analyzer	3-aminoisobutyrate was in the T2D top most significant metabolites from the total of 229 metabolites, sorted by pathway
	Kistner, S., et al. [90]	255 healthy women and men, urine samples	1H-NMR	15–30 min following an incremental cycling test, valine derivative 3-aminoisobutyrate increased
**Cluster 4**				
trans-4-hydroxy-L-proline	Kenéz, Á., et al. [91]	Twenty horses of various breeds, ages, and body weights	LC-MS/MS	Due to trans-4-hydroxy-l-proline, and high concentration of glycine, collagen has been shown to stimulate insulin secretion and stabilize blood sugar levels in individuals with T2D Lower plasma trans-4-hydroxyproline is associated with insulin dysregulation in horses
Glutamine	Floegel, A., et al. [19]	27,548 participants from the general population in Germany; 35–65 years of age	FIA-MS/MS	Glutamine improved insulin sensitivity
	Stancáková, A., et al. [92]	9369 non-diabetic or newly diagnosed T2D men from the population-based study (METSIM Study) (mean ± SD age, 57 ± 7 years; BMI, 27.0 ± 4.0 kg/m^2^)	1H-NMR	Elevated fasting and 2 h plasma glucose levels were associated with increasing levels of several amino acids and reduced levels of glutamine
	Palmer, N.D., et al. [93]	196 subjects European American, Hispanic, and African American Insulin Resistance Atherosclerosis Study (IRAS)	Mass spectrometry	the glutamine-to-glutamate ratio was found to predict insulin resistance after 5 years in participants
Hydroxyacetone	Reichard, G.A., et al. [67]	Patients with diabetes ketoacidosis (DKA)	Gas chromatograph equipped with a flame ionization detector	Acetol (1-hydroxyacetone), a possible metabolite of acetone, was detected in plasma of the patient during diabetic ketoacidosis
	Schumacher, D., et al. [94]	15 diabetes patients 15 healthy controls	LC-MS/MS	Diabetic patients without complications had the highest content of hydroxyacetone

## 4. Materials and Methods

### 4.1. Study Design

This is a multicenter cross-sectional study of patients with obesity and with different diabetes statuses who were scheduled for metabolic-bariatric surgery at the iHEAL Medical Centre KL, Sunway Medical Centre Velocity KL, Sunway Medical Centre Selangor, and KPJ Damansara Specialist Hospital, Malaysia. Anthropometric measurements were recorded and blood samples were collected pre-operatively.

Study participants were obese patients who were grouped into either obese only, prediabetes, or T2D. The diagnostic criteria were based on the HbA1c values [95] (Table 4). For comparison, healthy individuals (n = 26) were recruited as a control group.

This study involved 137 participants categorized into four groups: healthy controls (n = 26), obese only patients (n = 45), obese prediabetic patients (n = 37), and obese diabetic patients (n = 29). These participants except healthy controls were part of a cohort screened for bariatric surgery in multiple hospitals in the Klang Valley, Malaysia.

### 4.2. Individual Inclusion and Exclusion Criteria

Participants in the study were recruited based on the criteria outlined below.

- Inclusion Criteria

The inclusion criteria for the subjects are as follows: both men and women; age should be between 18 and 65 years; body mass index (BMI) ≥ 25 kg/m^2^ (Asian BMI: WHO/IASO/IOTF, 2000); and scheduled for metabolic surgery. Obese patients who fulfilled the criteria and have been diagnosed with prediabetes or T2D were also eligible. The inclusion criteria for healthy individuals are as follows: between 18 and 65 years of age; men and women; normal range BMI between 18.5 and 22.9 kg/m^2^; and without prediabetes, diabetes, or any related comorbidities. Written informed consent must be signed by all subjects.

- Exclusion Criteria

Patients and healthy individuals in the control group who were using medications that could impact their body weight at the time of recruitment, or who had medical conditions such as acromegaly, Cushing’s syndrome, heart failure, Crohn’s disease, or alcohol dependence, were excluded from the study. Individuals with a normal BMI of 18.5–22.9 kg/m^2^ but who present with comorbidities were also excluded.

### 4.3. Blood Biochemical Profile

Plasma samples (400 μL each) were collected from each participant. Before blood collection, the subjects were asked to fast overnight, which was estimated to be between 8 and 12 h. HbA1c was measured to determine the diabetes status. The blood samples were then centrifuged at 1500× *g* for 20 min. After centrifugation, the plasma was aliquoted into a few tubes and stored at −80 °C until further analysis. Lipid profiles were measured using chemistry analyzer.

### 4.4. Sample Preparation for Metabolomic Analysis

Two milliliters of blood were collected in lithium heparin tubes because other anticoagulants, such as EDTA and citrate, produce additional high-intensity signals in the NMR spectrum. The plasma samples were prepared according to published protocols for NMR metabolomic analysis [96]. This preparation ensured that the samples were ready for targeted and untargeted NMR metabolomics approaches.

### 4.5. Spectra Acquisition

The 1D ^1^H-NMR spectra were collected at a temperature of 26 °C using a JNM-ECZ-600 R 600 MHz spectrometer (JEOL, Tokyo, Japan). The combination of PRESAT and the Carr-Purcell-Meiboom-Gill (CPMG) pulse sequence was used to suppress water signals and broad protein resonances. NMR spectra with a spectral width of 12 ppm were acquired using 128 scans and a 660 s acquisition time [96]. After acquiring the NMR spectra, they were processed using the Chenomx NMR suite software, version 9.2.

### 4.6. Metabolite Identification

Chenomx NMR suite software, version 9.2 (Chenomx Inc., Edmonton, AB, Canada) was used to identify and quantify different groups’ metabolites by identifying the sections of the spectrum (ppm) responsible. This was accomplished by comparing the corresponding peak’s location, intensity, and linewidth with the 600 MHz Human Metabolome Database (HMDB) metabolites library. The area under the peak corresponded to the metabolites’ relative concentrations [96]. This study integrated targeted and untargeted NMR metabolomics, utilizing the advantages of both methodologies. The untargeted method offers a broad perspective of the metabolome, facilitating the identification of new biomarkers and metabolic pathways. The targeted method provides accurate quantification of specific metabolites, thereby improving the comprehension of their functions in metabolic processes. This integrated approach provides a comprehensive analysis of the metabolic changes linked to obesity, prediabetes, and diabetes. This dual approach enables a comprehensive understanding of the metabolic landscape and facilitates the identification of key biomarkers.

### 4.7. Data Pretreatment

The metabolomic data were analyzed using MetaboAnalyst 6.0 web application (https://www.metaboanalyst.ca/, (accessed on 15 August 2024), a comprehensive web-based tool suite for metabolomic data analysis. The raw data were uploaded to MetaboAnalyst 6.0 web application and subjected to data pretreatment. This included normalization to constant sum, data transformation using log normalization, and data scaling using Pareto scaling to ensure the data were properly prepared for downstream analysis.

### 4.8. Statistical Analysis

Several statistical and machine learning methods were employed using MetaboAnalyst 6.0 web application:

ANOVA for All Groups: One-way analysis of variance (ANOVA) was performed to identify significant differences among all groups. Post hoc analyses were also conducted to determine the specific group differences.

Fold Change Analysis Relative to Healthy Controls: Fold change analysis was used to compare the absolute value changes between the healthy control group and the other groups. This analysis helped identify metabolites that were significantly upregulated or downregulated relative to the healthy controls.

T-test Relative to Healthy Controls: T-tests were conducted to compare the metabolite levels between the healthy control group and each of the other groups. This analysis provided insights into the statistical significance of the differences observed.

OPLS-DA Relative to Healthy Controls: Orthogonal Partial Least Squares-Discriminant Analysis (OPLS-DA) was performed to identify metabolites that discriminated between the healthy control group and the other groups. This method helped in understanding the metabolic differences and identifying potential biomarkers.

### 4.9. Multivariate Analysis

PLS-DA VIP Score for All Groups: Partial Least Squares-Discriminant Analysis (PLS-DA) was used to identify the most important metabolites contributing to the separation between groups. The Variable Importance in Projection (VIP) scores were calculated to rank the metabolites based on their contribution to the model.

### 4.10. Clustering and Visualization

Hierarchical cluster analysis (HCA) and Heatmap: Hierarchical clustering was performed to group samples based on their metabolic profiles. Heatmaps were generated to visualize the clustering results and to identify patterns in the data.

Correlation Heatmap: Correlation analysis was conducted to identify significant correlations between metabolites. A correlation heatmap was generated to visualize these correlations, providing insights into the metabolic networks and relationships.

By using MetaboAnalyst 6.0 web application, we were able to perform a comprehensive analysis of the metabolomic data, including data pretreatment, statistical analysis, multivariate analysis, and visualization, which helped in identifying key metabolites and understanding the metabolic differences among the study groups.

### 4.11. Study Strengths

This study’s strengths lie in its comprehensive methodology, employing both targeted and untargeted NMR metabolomics approaches across multiple patient groups, and its use of advanced statistical analyses to identify key metabolites associated with obesity and diabetes.

### 4.12. Study Limitations

This study has some limitations that should be considered. First, the sample size is small, especially in the control group. However, we have sufficient samples for analysis [97]. Secondly, some unmeasured potential confounding factors (e.g., lifestyle or dietary intake) might have influenced our findings. Food intake influences metabolite levels, and gut microbiota plays a key role in modifying the metabolome and maintaining metabolic homeostasis [98]. Unfortunately, we do not have this data. However, the results based on diabetes status groups are a reasonable basis for diabetes exploration in metabolomics. Another limitation is the mean age difference between the healthy controls and obese groups. Several age-associated metabolites could reflect the changes in metabolisms during aging [99]. However, the younger, fitter group of healthy controls may be a good group to compare with. Hence, considering all limitations, our study results should be interpreted cautiously.

## 5. Conclusions

This study suggests that the use of ^1^H NMR metabolomics, in conjunction with conventional diabetes screening tools, could help to identify the mechanisms of diabetes and its progression by providing insights into underlying metabolic pathways. Identification of these metabolites may lead to more effective diabetes management for obese individuals. The metabolites identified in our study that may indicate the risk associated with T2D include fucose, 3-aminoisobutyrate, acetoacetate, and 3-hydroxybutyrate. These metabolites were significantly upregulated (by more than 2.6-fold) in obese patients with diabetes compared to healthy controls. This approach should incorporate precision medicine, utilizing genomic, and metabolomic biomarkers to personalize interventions, improve risk prediction, and initiate both lifestyle and pharmacological treatments.

Additionally, it is important to highlight those individuals classified as obese, specifically those with a BMI greater than 25 kg/m^2^—without prediabetes or diabetes according to Asian criteria—show significant divergences in metabolite levels compared to healthy controls. Therefore, more actions need to be taken at both community and individual levels to combat obesity and prevent diabetes-related comorbidities.

## Figures and Tables

**Table 1:** General characteristics of participants.

	Healthy Controls n = 26	Obese Only n = 45	Obese Prediabetes n = 37	Obese Diabetes n = 29
Age, years ^†^	25 (8)	37 (13)	41 (12)	38 (10)
**Age group, n (%)**				
<40 years	26 (100)	32 (71.1)	14 (37.8)	18 (62.1)
≥40 years		13 (28.9)	23 (62.2)	11 (37.9)
**Sex, n (%)**				
Female	16 (61.5)	36 (80.0)	19 (51.4)	16 (55.2)
Male	10 (38.5)	9 (20.0)	18 (48.6)	13 (44.8)
Weight, kg ^†^	55.42 (8.40)	97.91 (23.20) ^a^**	128.51 (41.55) ^a^**^,b^**	120.71 (29.92) ^a^**^,b^*
BMI, kg/m^2 †^	21.12 (2.02)	36.92 (7.80) ^a^**	46.09 (12.60) ^a^**^,b^**	43.47 (7.71)^a^**^,b^*
HbA1c, % ^†^	5.10 (0.58)	5.40 (0.40)	6.00 (0.35) ^a^**	6.90 (1.25) ^a^**^,b^**^,c^**
TC, mmol/L	5.00 (0.69)	5.22 (0.92)	4.94 (0.91)	5.58 (1.31)
TG, mmol/L ^†^	0.78 (0.34)	0.88 (0.66)	1.27 (0.69)	1.59 (0.79) ^a^**^,b^**
HDL-C, mmol/L ^†^	1.73 (0.40)	1.39 (0.34) ^a^**	1.25 (0.28) ^a^**	1.15 (0.19) ^a^**^,c^*
LDL-C, mmol/L	2.91 (0.51)	3.32 (0.81)	3.11 (0.84)	3.53 (1.15) ^a*^

Data are presented as mean (standard deviation), median (interquartile range) ^†^, or number of participants, n (%). A *p*-value of <0.05 was considered statistically significant. Comparisons were made as follows: ^a^ vs. Healthy control (** *p* < 0.001; * *p* < 0.05), ^b^ vs. Obese only (** *p* < 0.001; * *p* < 0.05); ^c^ vs. Obese prediabetes (** *p* < 0.001; * *p* < 0.05). TC; total cholesterol, TG; triglyceride, LDL-C; low density lipoprotein-cholesterol, HDL-C; high density lipoprotein-cholesterol.

**Table 2:** List of highly significant metabolites across study groups (*p* < 0.001).

No	Metabolites	Significance
1	Glycine	1.63 × 10^−13^
2	1,6-Anhydro-β-D-glucose	1.89 × 10^−12^
3	Glucose	1.73 × 10^−11^
4	Taurine	1.86 × 10^−11^
5	4-Hydroxyphenylacetate	4.56 × 10^−11^
6	myo-Inositol	5.69 × 10^−11^
7	Putrescine	6.51 × 10^−11^
8	π-Methylhistidine	1.39 × 10^−10^
9	3-Hydroxyphenylacetate	1.75 × 10^−10^
10	Trimethylamine N-oxide (TMAO)	1.63 × 10^−13^

**Table 4:** Diagnostic values for prediabetes and type 2 diabetes (T2D) based on HbA1c values (Malaysia Clinical Practice Guidelines, 2020) [95].

Category	HbA1c (%)
Normal	<5.7
Prediabetes	5.7–<6.3
Type 2 diabetes (T2D)	≥6.3

## Data Availability

The original contributions presented in this study are included in the article and Supplementary Materials. Further inquiries can be directed to the corresponding author.

## Supplementary Materials

The following supporting information can be downloaded at https://www.mdpi.com/article/10.3390/ijms26136216/s1.
