# Supplementary material for: Metabolomics: Uncovering Insights into Obesity and Diabetes
Source: Int J Mol Sci. 2025 Jun 27;26(13):6216. doi: 10.3390/ijms26136216 (PMC12249953; doi:10.3390/ijms26136216)
Supplement: Supplementary file 1 [file ijms-26-06216-s001.zip › ijms-3688042-supplementary.pdf]

**Supplementary information**

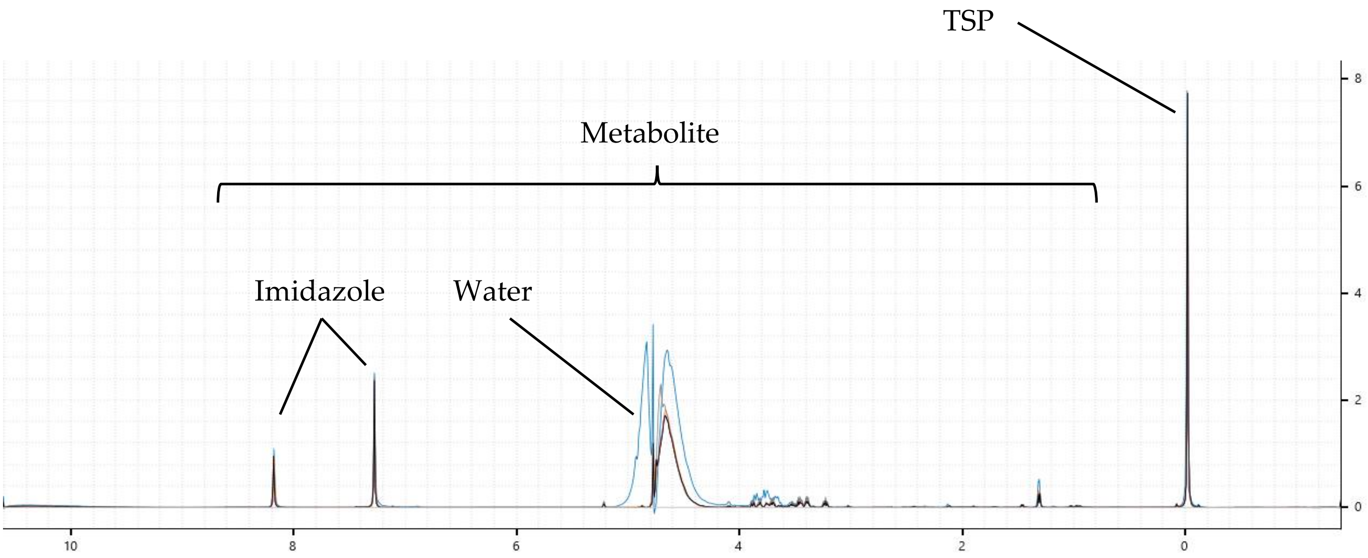


**Figure S1** Overlay of four spectra representing each group

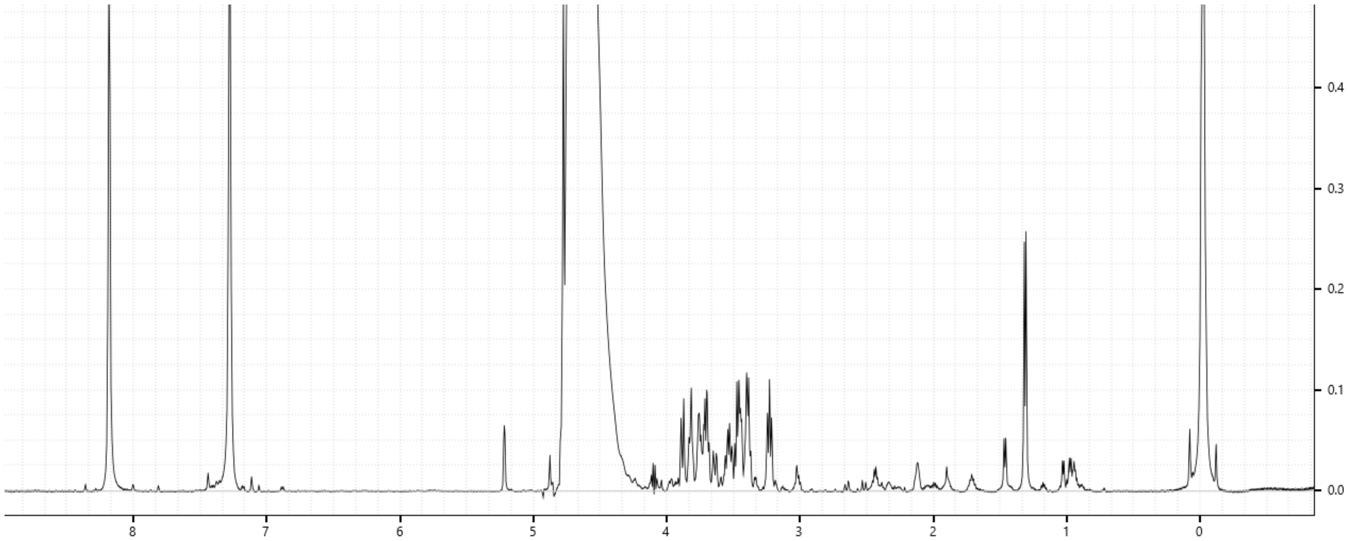


**Figure S2** Visualization of Metabolite Regions with Enhanced Scaling Parameters

Table S1. Metabolites list

| | Metabolites | Spectra Regions (ppm) |
|---|---|---|
| 1 | 1,6-Anhydro-β-D-glucose | 3.5, 3.7, 4.1, 4.6, 5.4 |
| 2 | 2-Methylglutarate | 1.1, 1.6, 1.7, 2.1, 2.2 |
| 3 | 2-Oxoglutarate | 2.4, 3.0 |
| 4 | 3-Aminoisobutyrate | 1.2, 2.6, 3.0, 3.1 |
| 5 | 3-Hydroxybutyrate | 1.2, 2.3, 2.4, 4.1 |
| 6 | 3-Hydroxyphenylacetate | 3.5, 6.8, 7.2 |
| 7 | 4-Hydroxyphenylacetate | 3.4, 6.9, 7.2 |
| 8 | Acetate | 1.9 |
| 9 | Acetoacetate | 2.3, 3.4 |
| 10 | Agmatine | 1.7, 3.0, 3.2, 7.2 |
| 11 | Alanine | 1.5, 3.8 |
| 12 | Anserine | 2.6, 2.7, 3.0, 3.2, 3.8, 4.5, 7.1, 8.2, 8.3 |
| 13 | Arginine | 1.6, 1.7, 1.9, 3.2, 3.8, 6.7, 7.2 |
| 14 | Cadaverine | 1.5, 1.7, 3.0 |
| 15 | Carnitine | 2.4, 3.2, 3.4, 4.6 |
| 16 | Choline | 3.2, 3.5, 4.1 |
| 17 | Citrate | 2.5, 2.7 |
| 18 | Creatinine | 3.0, 4.0 |
| 19 | Ethanolamine | 3.1, 3.8 |
| 20 | Fucose | 1.2, 3.4, 3.6, 3.7, 3.8, 3.9, 4.0, 4.1, 4.2, 4.5, 5.2, 5.3 |
| 21 | Gluconate | 3.7, 3.8, 4.0, 4.1 |
| 22 | Glucose | 3.2, 3.4, 3.5, 3.7, 3.8, 3.9, 4.6, 5.2 |
| 23 | Glutamate | 2.0, 2.1, 2.3, 2.4, 3.7 |
| 24 | Glutamine | 2.1, 2.4, 2.5, 3.8, 6.9, 7.6 |
| 25 | Glycine | 3.6 |
| 26 | Glycolate | 3.9 |
| 27 | Hippurate | 3.9, 7.5, 7.6, 7.8 |
| 28 | Histidine | 3.1, 3.2, 4.0, 7.1, 7.9 |
| 29 | Hydroxyacetone | 2.1, 4.4 |
| 30 | Isoleucine | 0.9, 1.0, 1.2, 1.5, 2.0, 3.7 |
| 31 | Lactate | 1.3, 4.1 |
| 32 | Leucine | 0.9, 1.0, 1.7, 3.7 |
| 33 | N-Methylhydantoin | 2.9, 4.1 |
| 34 | Ornithine | 1.7, 1.8, 1.9, 3.1, 3.8 |
| 35 | Proline | 2.0, 2.1, 2.3, 3.3, 3.4, 4.1 |
| 36 | Putrescine | 1.8, 3.0 |
| 37 | Pyruvate | 2.4 |
| 38 | Succinate | 2.4 |
| 39 | Taurine | 3.3, 3.4 |
| 40 | Threonine | 1.3, 3.6, 4.3 |
| 41 | Trimethylamine N-oxide | 3.3 |
| 42 | Tyrosine | 3.0, 3.2, 3.9, 6.9, 7.2 |
| 43 | Valine | 1.0, 2.3, 3.6 |
| 44 | *cis*-aconitate | 3.1, 5.7 |
| 45 | *myo*-inositol | 3.3, 3.5, 3.6, 4.1 |
| 46 | *trans*-4-hydroxy-L-proline | 2.1, 2.4, 3.4, 3.5, 4.3, 4.7 |
| 47 | π-methylhistidine | 3.2, 3.3, 3.7, 4.0, 7.1, 8.0 |
